# Establishment and clinical application of a droplet digital PCR method for the detection of *Edwardsiella tarda*

**DOI:** 10.3389/fvets.2024.1439743

**Published:** 2024-09-06

**Authors:** Min Li, Xiaojun Li, Yifei Ye, Jinfang Yin, Zuanlan Mo, Haiyan Xie, Yanqiu Zhu, Liangning Zhong, Xianpeng Zhang, Junlong Bi

**Affiliations:** ^1^College of Veterinary Medicine, Yunnan Agricultural University, Kunming, China; ^2^Dongguan Center for Animal Disease Prevention and Control, Dongguan, Guangdong, China; ^3^Dongguan Key Laboratory of Zoonosis, Dongguan, Guangdong, China

**Keywords:** *Edwardsiella tarda*, droplet digital PCR, linear relationship, specificity, clinical application

## Abstract

*Edwardsiella tarda* (*E. tarda*) can infect humans and a variety of animals, including fish, amphibians, reptiles, birds, and mammals. However, a more highly sensitive, specific, and repeatable test for its detection is lacking. The objective of this study was to develop a highly sensitive, specific, and repeatable droplet digital polymerase chain reaction (ddPCR)-based method for the quantitative detection of *E. tarda*. The *gyrB* gene was selected as the target gene, and primers and probe were designed and synthesized. Using *E. tarda* genomic DNA as templates, the reaction method was optimized to establish a linear relationship with real-time PCR detection methods. The sensitivity, specificity, and repeatability of the method were analyzed, and clinical samples were tested. When the primer and probe concentrations were 900 and 300 nM, respectively, and the annealing temperature was 57°C, the efficiency of the ddPCR amplification reaction was highest and the boundary between positive and negative droplet distribution was clearest. The sensitivity was high, with detection limit being as low as 0.56 copies·μL^−1^; additionally, and a good linear relationship (*R*^2^ = 0.9962) between ddPCR and real-time PCR detection, within the range of 1–25,000 copies·μL^−1^, was evident. The repeatability was good, with a detection coefficient of variation of 2.74%. There was no cross-reactivity with 15 other common pathogenic microorganisms in aquatic animals (*Streptococcus agalactiae*, *Streptococcus iniae*, *Streptococcus suis* type 2, *Nocardia seriolae*, *Vibrio parahaemolyticus*, *Aeromonas sobria*, red sea bream iridovirus, decapod iridescent virus 1, enterocytozoon hepatopenaei, carp edema virus, Koi herpesvirus, goldfish hematopoietic necrosis virus, tilapia lake virus, viral nervous necrosis virus, or grass carp reovirus) in positive samples. Among the 48 clinical samples, including *Bahaba taipingensis* and its live food fish, pond water samples, and routine monitoring samples (Koi), 21 were positive for *E. tarda*, consistent with the bacterial isolation and identification results. The *E. tarda* ddPCR detection method has high specificity, sensitivity, and repeatability, can more accurately quantify *E. tarda*, and provides a useful reference for research related to this bacterium.

## 1 Introduction

*Edwardsiella tarda* (*E. tarda*) is a Gram-negative bacillus that was first isolated from the Japanese eel (*Anguilla japonica*) by Hoshina in 1962 (1). *E. tarda* has a wide range of hosts, can infect aquatic animals (2–4) and birds (5), and has been isolated from the feces of South China tigers (*Panthera tigris* ssp. *amoyensis*) (6). Infection by this bacterium can occur in all seasons; the higher the water temperature, the longer the disease cycle and the greater the damage. Infection presents mainly as skin hemorrhage, ascites, hepatosplenic and renal swelling, congestion, and hemorrhagic sepsis (7, 8). The geographical scope of these infections has been expanding, with reports of severe economic losses to the aquaculture industry in the United States (9), Germany (10), Italy (11), and South Africa (12). *E. tarda* has been reported to cause disease in various economically important fish species, including Chinese soft-shelled turtles, carp, tilapia, flounder, and turbot (1, 2, 13), have been a matter of concern for the Chinese aquaculture industry since 1989 (2).

*Edwardsiella tarda* is an important zoonotic bacterium. Exposure to water contaminated with *E. tarda* or undercooked, infected food causes symptoms such as low-grade fever, gastroenteritis, liver abscess, meningitis, and sepsis and even lead to death (14–16). Therefore, it is important to establish a method for detecting *E. tarda* with high specificity and sensitivity and good reproducibility to ensure public health safety and prevent and control aquatic animal diseases.

Research on the detection of *E. tarda* initially focused on traditional bacterial isolation and culture methods (17), followed by enzyme staining (18), enzyme-linked immunosorbent assay (ELISA) (19, 20), PCR (21), real-time PCR (22), loop-mediated isothermal amplification (LAMP) (23, 24), and gene chips (25). However, a more highly-sensitive, specific, and repeatable test is still lacking. The objective of the current study was to develop a droplet digital PCR (ddPCR)-based method for detecting *E. tarda* to provide technical support for the prevention and treatment of infections caused by *E. tarda*.

## 2 Materials and methods

### 2.1 Standards and training sources

*Edwardsiella tarda* (DG20230920, Dongguan, China) was isolated and identified at the Laboratory of the Dongguan Animal Disease Prevention and Control Center. *E. tarda* was aseptically collected from the spleen, liver, and kidney of *Bahaba taipingensis*, and inoculated onto tryptone soybean Agar (TSA) plate for 24 h at 30°C. *Streptococcus iniae* (ATCC29178, American, Virginia) was obtained from the American Type Culture Collection (ATCC). *Streptococcus agalactiae*, *Nocardia seriolae*, *Vibrio parahaemolyticus*, *Aeromonas sobria*, and *Streptococcus suis* type 2 were preserved in the Dongguan Animal Disease Prevention and Control Center laboratory (Dongguan, China). Nucleic acids positive for viral nervous necrosis virus (VNNV), grass carp reovirus (GCRV), tilapia lake virus (TiLV), red sea bream iridovirus (RSIV), decapod iridescent virus 1(DIV1), and enterocytozoon hepatopenaei (EHP) were obtained from the Guangdong Provincial Center for Animal Disease Prevention and Control (Guangzhou, China). Nucleic acids of carp edema virus (CEV) were obtained from the Beijing Aquatic Technology Extension Station (Beijing, China). Nucleic acids of koi herpesvirus (KHV) and goldfish hematopoietic necrosis virus (GFHNV) were obtained from the Chinese Academy of Inspection and Quarantine (Beijing, China).

### 2.2 Sample collection

A total of 48 clinical samples were collected from the rescue base of the Dongguan *Bahaba Taipingensis* Nature Reserve (*Bahaba Taipingensis*, live food fish, and pond water).

### 2.3 Design of primers and probe

The whole gene sequence was accessed from the GenBank (Accession number: MG026726.1). Given that *gyrB* is more suitable for distinguishing and identifying *E. tarda, gyrB* was selected as the target gene (26). The primer and probe sequences are detailed in Table 1.

**Table 1:** Primers and probe for detecting *E. tarda* nucleic acid using ddPCR and real-time PCR.

Primer, probe	Sequences (5′–3′)
Upstream primer	AGCGATGCACGTGAGGTT
Downstream primer	TTAGTCTGCGAGGAGAACTTG
Probe	FAM-CACCTTCACAGATACCACGGCGAT-BHQ1

### 2.4 DNA extraction

DNA was extracted from the liver of *Bahaba Taipingensis* (or its forage fish) using a DNA Kit (Tiangen, Beijing, China) according to the manufacturer’s instructions, the initial concentration of DNA obtained was 2.5*10^6^ copies/L. The DNA of *E. tarda* was diluted 1,000-fold with double-distilled water (dd-H_2_O). This was followed by four-fold serial dilutions, with eight dilutions for the sensitivity analysis; the third dilution was selected for the repeatability analysis (Table 2).

**Table 2:** Nucleic acid concentrations used for ddPCR in *E. tarda.*

Nucleic acid concentrations	Code	Concentration proportionality
Initial concentration	A	
Sensitivity analysis test first concentration	B1	/1000
Sensitivity analysis test follow-up concentrations	B2-B8	/4

### 2.5 Development of ddPCR-based detection method for *Edwardsiella tarda*

The *E. tarda* ddPCR reaction system consisted of 2 × ddPCR Supermix for probe, upstream and downstream primers, probe, dd-H_2_O, and template. The 20 μL ddPCR reaction-generated droplets were transferred to a 96-well plate, placed in a thermal cycler for amplification, and then placed in a droplet reader to analyze the results. The ddPCR reaction system and procedures are listed in Table 3.

**Table 3:** Reaction system and reaction program for *E. tarda* ddPCR.

Reaction system	Dosage	Reaction procedure		
		Temperature (°C)	Time	Number of cycles
2 × ddPCR Supermix^TM^ for probes	10	95	10 min	1
Upstream primer	Adjustment to different reaction concentrations	94	30s	40
Downstream primer		Annealing temperature adjusted according to test program	60s	
Probe				
DNA template	2.0	98	10 min	1
dd-H_2_O	Supplement to 20 μL	4	∞	

A 1,000-fold dilution gradient of *E. tarda* nucleic acids was used as a template to optimize the ddPCR conditions, including primers, probe concentration, and annealing temperature; each factor was set at five levels (Table 4). The SPSS software (version 16.0) was used to design an orthogonal array of optimization factors, arranged according to the annealing temperature, from small to large, for a total of 25 test protocols (Table 5), and each test protocol had three replicates. The main strategy for optimization was to maximize the difference in the fluorescence amplitude between the negative and positive droplet partitions and minimize the number of partitions with moderate fluorescence intensity.

**Table 4:** Optimization factors and their levels for ddPCR in *E. tarda.*

Optimization factor		Response level				
	Code	1	2	3	4	5
Primer concentration (nM)	A	300	500	700	900	1,100
Probe concentration (nM)	B1	100	150	200	250	300
Annealing temperature (°C)	B2-B8	51	53	55	57	59

**Table 5:** Orthogonal array design and advantages and disadvantages of the optimization of reaction conditions for *E. tarda* ddPCR.

Program number	Primer concentration (nM)	Probe concentration (nM)	Annealing temperature (°C)	Advantages and disadvantages*
1	300	200	51	15
2	500	250	51	14
3	700	300	51	8
4	900	100	51	6
5	1,100	150	51	7
6	300	100	53	5
7	500	150	53	4
8	700	200	53	3
9	900	250	53	2
10	1,100	300	53	1
11	300	250	55	20
12	500	300	55	21
13	700	100	55	9
14	900	150	55	13
15	1,100	200	55	17
16	300	150	57	16
17	500	200	57	18
18	700	250	57	22
19	900	300	57	25
20	1,100	100	57	11
21	300	300	59	24
22	500	100	59	10
23	700	150	59	12
24	900	200	59	19
25	1,100	250	59	23

*Scores were determined between the best (25 points) and worst (1 point) based on the difference in fluorescence amplitude between negative and positive microdroplet partitions and minimizing the number of partitions with moderate fluorescence intensity.

### 2.6 Establishment of standard curves

After 1,000-fold dilution of *E. tarda* strain nucleic acid with dd-H_2_O, a four-fold serial dilution was performed for eight gradients and three replicates of each gradient were included. The ddPCR and real-time PCR-based detection was performed simultaneously. Negative and blank controls were used. The logarithmic value of the number of *E. tarda* DNA molecules measured using ddPCR was used as the abscissa, and the cycle threshold (*C*t) value of real-time PCR was used as the ordinate for constructing the standard curve. The primers, probe sequences, reaction systems, and real-time PCR reaction conditions are shown in Tables 1, 6.

**Table 6:** Real-time fluorescent PCR reaction system and reaction program for *E. tarda* DNA.

Reaction system	Dosage (μL)	Protocol		
		Temperature (°C)	Time	Number of cycles
5 × PCR buffer	5	95	3 min	1
dNTPs	2			45
*Taq* DNA polymerase	0.6	95	15 s	
Primer/probe	1.25/1			
DNA template	5	55 (fluorescence collection)	30 s	
dd-H_2_O	8.9			

### 2.7 Specificity test

The DNA or cDNA of *S. agalactiae*, *S. iniae*, *S. suis* type 2, *N. seriolae*, *V. parahaemolyticus*, *A. sobria*, RSIV, DIV1, EHP, CEV, KHV, GFHNV, VNNV, ISKNV, TiLV, and GCRV were tested to evaluate the specificity of ddPCR for *E. tarda*.

### 2.8 Reproducibility test

The nucleic acids of the *E. tarda* strain were diluted 1,000-fold with dd-H_2_O, followed by a four-fold serial dilution for a total of eight gradients. The third dilution of DNA was detected according to the (optimized item 1.2.4), and 13 replicates were used to calculate the coefficient of variation to evaluate the stability of the *E. tarda* ddPCR (Table 7).

**Table 7:** Within-group reproducibility test for ddPCR of B3 retarded *E. tarda’*s.

Sample code	Number of cDNA molecules
	Normalized detected target DNA/(copy-μL^−1^)
*E. tarda-*1	1,080
*E. tarda-*2	1,050
*E. tarda-*3	1,100
*E. tarda-*4	1,100
*E. tarda-*5	1,090
*E. tarda-*6	1,160
*E. tarda-*7	1,080
*E. tarda-*8	1,080
*E. tarda-*9	1,110
*E. tarda-*10	1,130
*E. tarda-*11	1,090
*E. tarda-*12	1,150
*E. tarda-*13	1,100
Mean ± SD	1101.54 ± 30.23
CV (%)	2.74

### 2.9 Testing of clinical samples

The nucleic acids of 48 samples of *Bahaba taipingensis*, live food fish, and ponds water were detected using the established *E. tarda* ddPCR method, and the results were compared with those obtained using real-time PCR and standard bacterial isolation and culture identification methods (Table 8).

**Table 8:** Detection of *E. tarda* ddPCR, real-time PCR, and three methods of bacterial isolation and culture identification of clinical samples.

Clinical samples		Results of different testing methods		
Number	Designation	ddPCR (number of microtitres)	Real-time PCR (*C*t)	Bacterial isolation and culture identification
1	*Bahaba taipingensis* liver-1	6,540	20.24	+
2	*Bahaba taipingensis* liver-2	1,592	22.28	+
3	*Bahaba taipingensis* liver-3	1,595	22.27	+
4	*Bahaba taipingensis* liver-4	1,476	22.38	+
5	*Bahaba taipingensis* liver-5	270	24.83	+
6	*Cirrhinus molitorella*-1	25	UNDET	+
7	*Cirrhinus molitorella*-2	35	27.78	+
8	*Micropterus salmoides*-1	21	UNDET	+
9	*Micropterus salmoides*-2	21	UNDET	+
10	*Micropterus salmoides*-3	17	UNDET	+
11	*Micropterus salmoides*-4	0	UNDET	−
12	*Micropterus salmoides*-5	0	UNDET	−
13	Ponds water-1	8	UNDET	+
14	Ponds water-2	0	UNDET	−
15	Ponds water-3	0	UNDET	−
16	Ponds water-4	0	UNDET	−
17	Ponds water-5	0	UNDET	−
18	Ponds water-6	0	UNDET	−
19	Ponds water-7	0	UNDET	−
20	Ponds water-8	0	UNDET	−
21	*Cirrhinus molitorella*-3	0	UNDET	−
22	*Cirrhinus molitorella*-4	0	UNDET	−
23	*Micropterus salmoides*-6	0	UNDET	−
24	*Micropterus salmoides*-7	0	UNDET	−
25	*Micropterus salmoides*-8	0	UNDET	−
26	*Micropterus salmoides*-9	0	UNDET	−
27	*Micropterus salmoides*-10	0	UNDET	−
28	*Micropterus salmoides*-11	0	UNDET	−
29	*Micropterus salmoides*-12	0	UNDET	−
30	*Ctenopharyngodon idellus*	0	UNDET	−
31	*Squaliobarbus curriculus*-1	0	UNDET	−
32	*Squaliobarbus curriculus*-2	0	UNDET	−
33	*Squaliobarbus curriculus*-3	0	UNDET	−
34	*Bahaba taipingensis* liver-6	1,433	22.43	+
35	*Bahaba taipingensis* liver-7	1,213	22.67	+
36	*Bahaba taipingensis* kidney-1	1,077	22.84	+
37	*Bahaba taipingensis* kidney-2	1,294	22.57	+
38	*Bahaba taipingensis* kidney-3	1,540	22.32	+
39	*Bahaba taipingensis* kidney-4	223	25.11	+
40	*Bahaba taipingensis* Spleen-1	1,483	22.38	+
41	*Bahaba taipingensis* Spleen-2	1,523	22.34	+
42	*Bahaba taipingensis* Spleen-3	1,604	22.26	+
43	*Bahaba taipingensis* Spleen-4	1754	22.14	+
44	Daily monitoring samples (*Cyprinus carpio*-1)	0	UNDET	−
45	Daily monitoring samples (*Cyprinus carpio*-2)	0	UNDET	−
46	Daily monitoring samples (*Cyprinus carpio*-3)	0	UNDET	−
47	Daily monitoring samples (*Cyprinus carpio*-4)	0	UNDET	−
48	Daily monitoring samples (*Cyprinus carpio*-5)	0	UNDET	−

+ indicates positive; − indicates negative.

### 2.10 Statistical analyses

The ddPCR data for *E. tarda* were used for image processing and analysis using the Quantasoft software (26). A total number of droplets >10,000 was used as the criterion for ddPCR. No positive droplets were detected in the negative or blank controls, indicating that the system was not contaminated or specifically amplified. The key strategy for optimization was to maximize the difference in the fluorescence amplitude between the negative and positive droplet partitions and minimize the number of partitions with moderate fluorescence intensity. After PCR amplification of all the droplets, the droplets containing the target were amplified, and the droplets with higher fluorescence intensity were judged as positive droplets; the droplets that did not contain the target were not amplified and those with lower fluorescence intensity were considered to be negative droplets.

After the droplet reader interpretation, the droplet population would have a positive rate value, *P*. Upon combining this result with the Poisson distribution algorithm, the copy number of each positive droplet would be −ln (1 − *p*), and the concentration (copies μL^−1^) of the sample could be converted to a fixed and known volume of each droplet. Experimental data are presented as the mean ± standard deviation (X ± SD).

Number of DNA molecules = number of copies per microliter (copies·μL^−1^) × 20 μL ddPCR reaction system/DNA template dosage.

## 3 Results

### 3.1 Establishment of a ddPCR method for the detection of *Edwardsiella tarda*

**Figure 1:**
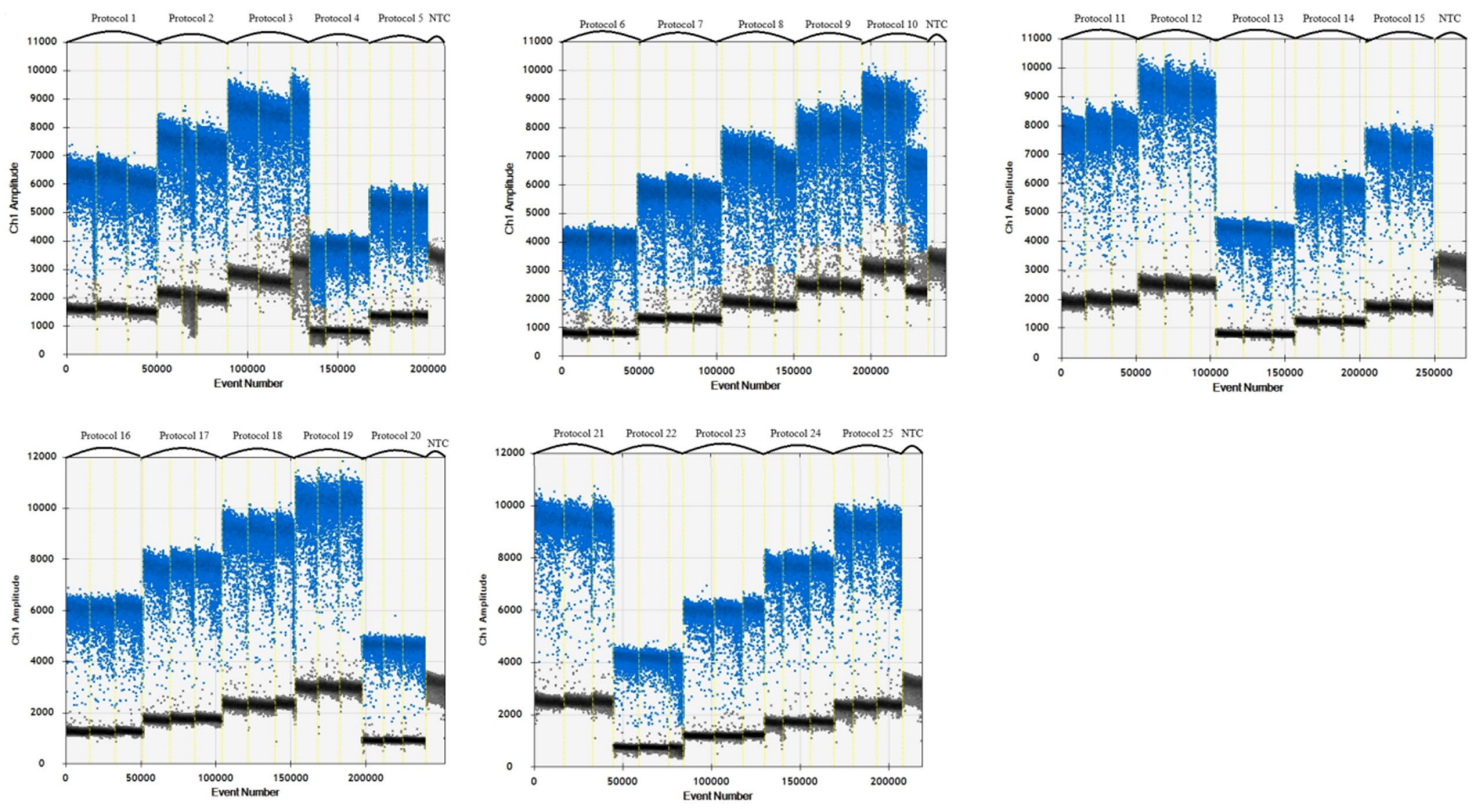
Primer, probe and annealing temperature optimization plots.

When the primer concentration was 900 nM, probe concentration was 300 nM, and annealing temperature was 57°C, the resultant fluorescence intensity was the highest, the amplification reaction had the highest efficiency, and the boundary between the distribution of positive and negative droplets was most obvious. Test scheme 19 (primer concentration, probe concentration, and annealing temperature of 900 nM, 300 nM, 57°C, respectively) was therefore close as the optimal permutation of conditions (Table 5 and Figure 1).

### 3.2 Sensitivity tests and construction of standard curves

**Figure 2:**
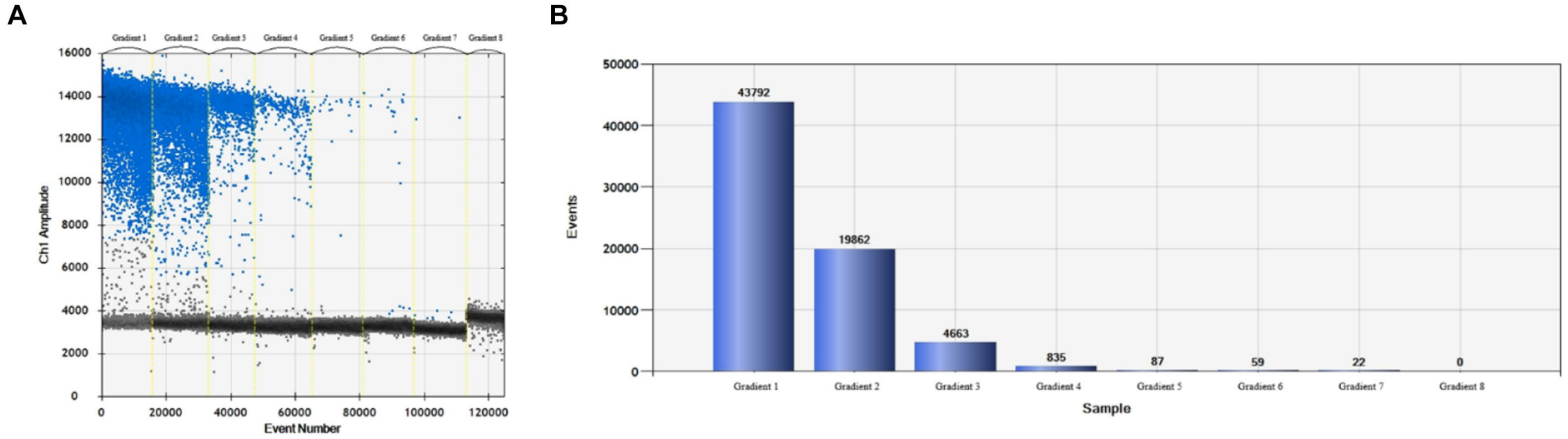
*Edwardsiella tarda* ddPCR sensitivity test. **(A)** Microdroplet 1D, **(B)** microdroplet histogram.

**Figure 3:**
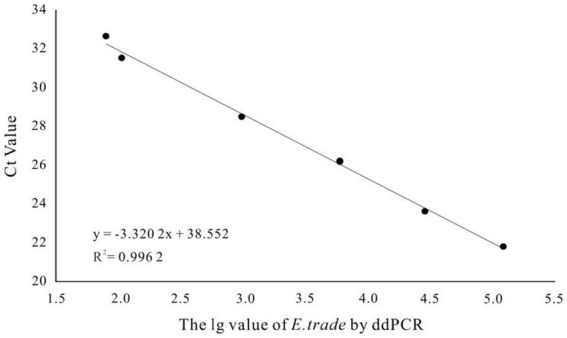
*Edwardsiella tarda* ddPCR and real-time PCR linear relationship.

More than 13,000 results of the ddPCR tests on the DNA of the eight strains of *E. tarda* with different concentration gradients were obtained. The average lower limit of ddPCR detection was 0.56 copies·μL^−1^ (Figure 2). The log value of the molecular number of *E. bradynia* DNA measured using ddPCR was used as the abscissa, and the *C*t value of real-time PCR was used as the ordinate to construct the standard curve. The gradients of ddPCR showed excellent correlation over the detection range, *y* = −3.3202*x* + 38.552, *R*^2^ = 0.9962. This standard curve was used to calculate the number of nucleic acid molecules in a clinical sample (Table 9 and Figure 3).

**Table 9:** Sensitivity test of the ddPCR and real-time PCR for *E. trade.*

	ddPCR				Real-time PCR			
Sample	Total droplets	Positive droplets	Normalized detected target DNA/(copies·μL^−1^)	Mean ± standard deviation	CV	Ct	Mean ± standard deviation	CV
*E. trade*-1	15,969	14,026	2,478			22.04		
	16,386	14,194	2,367	2457.67 ± 82.4	3.35	21.4	21.77 ± 0.33	1.53
	17,627	15,572	2,528			21.88		
*E. trade*-2	16,879	6,540	577			23.62		
	17,284	6,734	581	576.33 ± 5.03	0.87	23.78	23.57 ± 0.24	1.01
	17,143	6,588	571			23.31		
*E. trade*-3	17,299	1,592	114			25.99		
	16,502	1,595	120	120.33 ± 6.51	5.41	26.46	26.16 ± 0.26	1.00
	14,411	1,476	127			26.03		
*E. trade*-4	16,310	270	19.6			28.52		
	17,686	309	20.7	19.57 ± 1.15	5.88	28.08	28.45 ± 0.34	1.21
	16,475	256	18.4			28.76		
*E. trade*-5	15,862	25	1.9			30.46		
	16,790	27	1.9	2.13 ± 0.4	18.94	30.53	30.57 ± 0.13	0.44
	16,151	35	2.6			30.72		
*E. trade*-6	15,644	21	1.6			33.31		
	13,917	21	1.8	1.6 ± 0.2	12.50	32.45	32.85 ± 0.43	1.32
	14,616	17	1.4			32.8		
*E. trade*-7	16,443	8	0.46			35.72		
	15,488	6	0.65	0.56 ± 0.11	18.54	34.84	35.1 ± 0.54	1.53
	14,088	8	0.57			34.75		
*E. trade*-8	15,577	0	0			35.01		
	16,439	0	0	–	–	–	–	–
	16,282	0	0			36.41		

### 3.3 Specificity test

**Figure 4:**
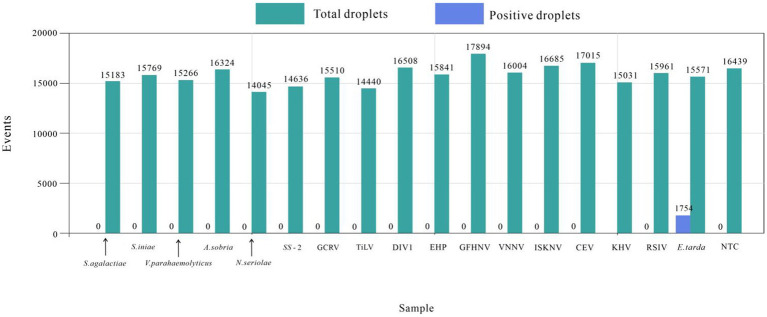
*Edwardsiella tarda*’s ddPCR specificity test.

The established ddPCR method for *E. tarda* was used to test the DNA or cDNA of *S. agalactiae*, *S. iniae*, *S. suis* type 2, *N. seriolae*, *V. parahaemolyticus*, *A. sobria*, RSIV, DIV1, EHP, CEV, KHV, GFHNV, VNNV, ISKNV, TiLV, and GCRV. The number of droplets amplified by ddPCR was more than 14,000, and the results were valid. Except for the *E. tarda*-positive samples (1,754), none of the other 16 viral nucleic acid tests showed positive droplets. These findings indicated that the developed ddPCR method had good specificity for *E. tarda* (Figure 4).

### 3.4 Reproducibility test

**Figure 5:**
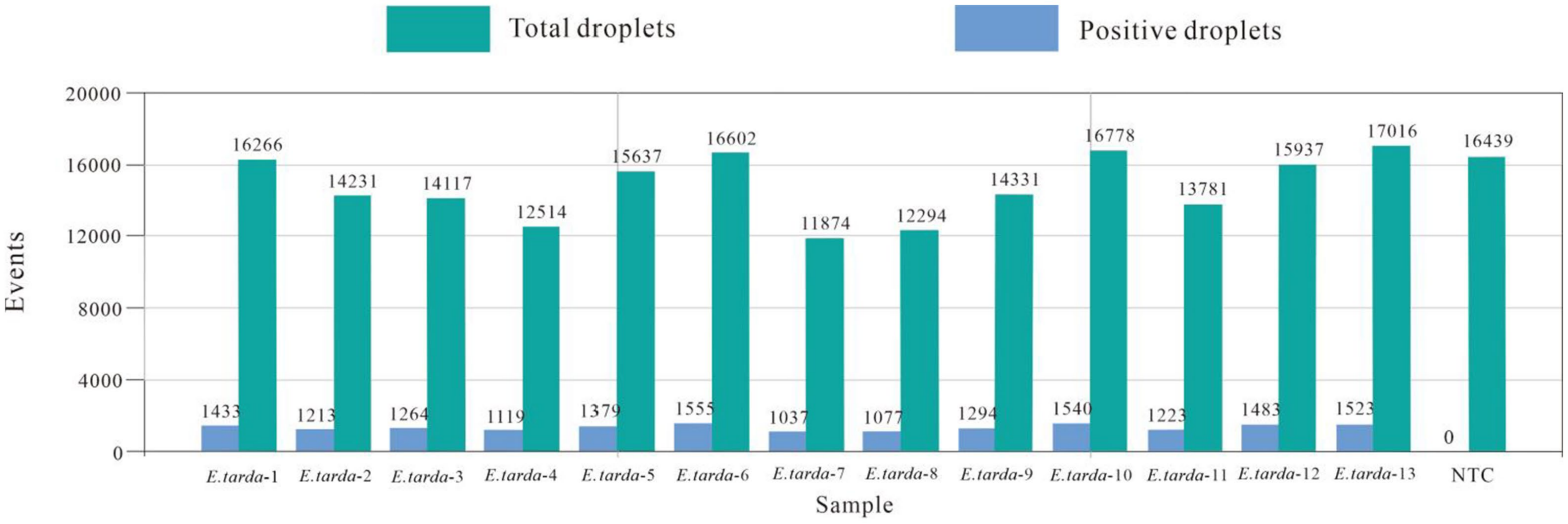
Within-group reproducibility test for ddPCR.

The ddPCR detected nucleic acids at the B3 dilution with 13 replicates. The number of droplets amplified by ddPCR was more than 11,000. The coefficient of variation (CV) of the within-group assay was 2.74%, demonstrating that the established ddPCR for *E. tarda* detection had favorable reproducibility (Table 7 and Figure 5).

### 3.5 Test results of clinical samples

**Figure 6:**
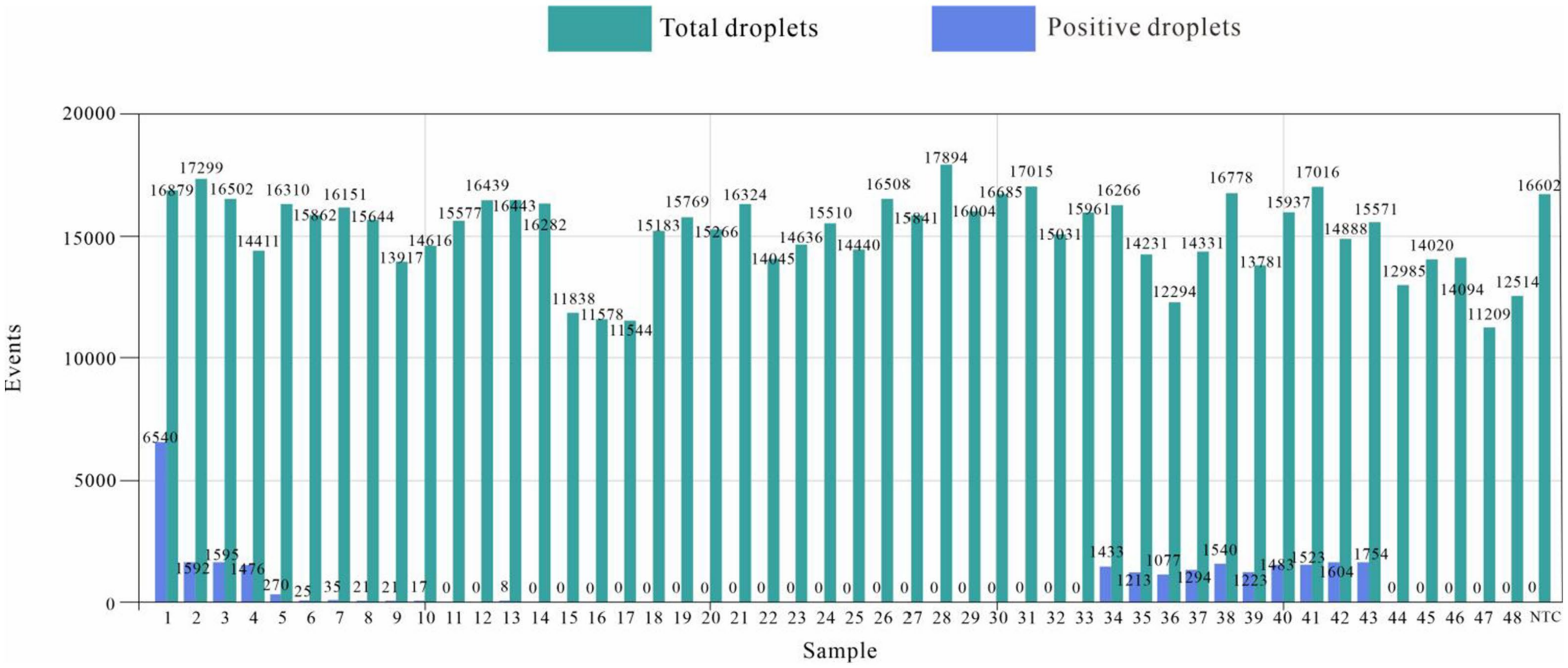
*Edwardsiella tarda* ddPCR clinical sample results.

The number of droplets amplified via ddPCR was more than 11,000, and the results were positive; 21 samples were positive for *E. tarda*, and the remaining 27 samples were negative for *E. tarda* nucleic acid (Figure 6). However, sample 6, 8, 9, 10, and 13 were not confirmed to be positive for *E. tarda* nucleic acids by 16S rRNA or real-time PCR after 24 h of incubation in Trypticase Soy Broth liquid medium (Table 8). The ddPCR-based method for *E. tarda* detection was feasible and more sensitive than quantitative PCR and was suitable for detection in clinical samples as well as for calibrating standard *E. tarda* samples.

## 4 Discussion

Edwards is a collective term for aquatic animal diseases caused by *E. tarda*. The genus *Edwardsiella* includes three species- *Edwardsiella ictaluri*, *Edwardsiella hoshinae*, and *E. tarda* (14). There have been reports of more than 20 species of aquatic animals, reptiles, and other animals infected by *E. tarda*, which has caused huge economic losses to the aquaculture industry. More importantly, *E. tarda* is the only species in the genus *Edwardsiella* that infects humans (27), posing a serious threat to public health and safety. Antibiotics are predominantly used to prevent and control aquatic bacterial diseases caused due to the non-standard and unscientific use of drugs in fisheries. In recent years, aquatic product quality and safety incidents have occasionally occurred, and the resistance of pathogenic bacteria to drugs has gradually increased. This has created challenges for the prevention and control of diseases.

In the early stage of *E. tarda* infection in aquatic animals, the main manifestations are external, such as individual congestion or hemorrhage, anal redness, swelling, and protrusion (8, 9), which cannot be detected in time because of the particularity of the living environment. The onset of the disease is acute, and morbidity and mortality rates are high (28). Therefore, it is important to develop a highly sensitive and specific detection method to prevent and control the infections caused by *E. tarda*. Currently, the diagnosis of *E. tarda* includes characterization and observation of diseased aquatic animals, isolation and culture of bacteria, serological testing, and molecular biological testing. Characterization and observation cannot accurately confirm *E. tarda* infection in aquatic animals; therefore, further testing is required. Bacterial isolation and culture assays are labor-intensive, time-consuming, and do not allow timely treatment or control of infections. Molecular biological detection methods include PCR based on 16S rRNA, qPCR, and LAMP, based on the hemolysin gene. Ordinary PCR detection is cumbersome, with a risk of EB contamination. The qPCR-based detection cannot achieve absolute quantification.

The ddPCR is a next-generation technology based on real-time PCR that can be used for absolute quantification of nucleic acids of interest. In the droplet generator, the reaction system is separated into 10,000 ~ 20,000 small water-in-oil droplets that act as PCR bioreactors. After conventional PCR amplification, each reaction chamber contains zero to multiple copies of the nucleic acid of interest. The droplets are analyzed separately using a droplet reader similar to the flow cytometry for fluorescence. The Poisson distribution is used to determines the copy number. This technique has been applied to detect aquatic animal diseases (29). In the current study, specific primers and probe were designed, and the ddPCR system and amplification program were optimized, providing a new theoretical and practical means for the rapid, accurate, and sensitive detection of diseased by *E. tarda*.

In the current study, ddPCR detection technology was used for the first time to detect *E. tarda*. The *gyrB* gene, suitable for the differentiation and identification of strains, was selected as the target gene (30) and applied to the detection of clinical samples. The minimum detection limit of the ddPCR method for *E. tarda* was 0.56 copies·μL^−1^, which was higher than that of other molecular biological detection methods reported (22, 31). Sun et al. (22) used nested PCR to detect *E. tarda* at least 10 fg of *E. tarda*, but this approach was only suitable for quantitative detection using multiple dilutions when the detection object was known. Li et al. (32) combined recombinase polymerase amplification (RPA) technology with a lateral flow strip (LFS) to establish an RPA-LFS method for the detection of *E. tarda*; 1 × 10^1^ CFU/g was the lowest detection amount, but this concentration of bacteria needed to be enriched and cultured for 4 h before being detected.

Chen and Lai (24) established a method for detecting *E. tarda* using LAMP; however, false positives were observed. The ddPCR method established in the current study for detecting *E. tarda* was highly specific. It showed no cross-reactivity with 16 microorganisms, including *S. agalactiae*, *S. iniae*, *S. suis* type 2, *N. seriolae*, *V. parahaemolyticus*, *A. sobria*, RSIV, DIV1, EHP, CEV, KHV, GFHNV, VNNV, ISKNV, TiLV, and GCRV. The DNA from *E. tarda* was used as the template. When the primer concentration was 900 nmol·L^−1^, probe concentration was 300 nmol·L^−1^ and annealing temperature was 57°C, the distribution boundary of positive and negative droplets in the ddPCR amplification reaction was the most obvious. The coefficient of *E. tarda* established in this study was 2.74%, which demonstrates good stability. The lowest detection limit was 0.56 copies·μL^−1^ in the range of 1–25,000 copies·μL^−1^.

## 5 Conclusion

The *E. tarda* ddPCR established in this study exhibits high specificity, high sensitivity and good reproducibility and can be used for clinical diagnosis in the early stages of *E. tarda* infection in aquatic animals. Application to testing in reptiles and humans can be investigated subsequently. This study provides technical support for the early detection of *E. tarda* infections.

## Data availability statement

The original contributions presented in the study are included in the article/supplementary material, further inquiries can be directed to the corresponding authors.

## Ethics statement

The animal study was approved by Yunnan Agricultural University. The study was conducted in accordance with the local legislation and institutional requirements.

## Author contributions

ML: Writing – original draft. XL: Writing – original draft, Data curation. YY: Writing – original draft, Data curation, Writing – review & editing. JY: Conceptualization, Writing – review & editing. ZM: Investigation, Writing – review & editing. HX: Writing – original draft. YZ: Writing – original draft. LZ: Writing – original draft, Methodology. XZ: Writing – original draft. JB: Writing – review & editing, Data curation, Supervision, Writing – original draft.
